# Localizing the epileptogenic zone in drug-resistant epilepsy based on ictal SEEG high-resolution time-frequency representation

**DOI:** 10.3389/fnins.2026.1891224

**Published:** 2026-08-04

**Authors:** Genyu Fu, Shijie Chen, Danni Yang, Sixian Chan, Yalin Wang, Zhe Shi, Qiang Li

**Affiliations:** 1 School of Information Science and Engineering, Lanzhou University, Lanzhou, China; 2 School of Materials Science and Engineering, Lanzhou University of Technology, Lanzhou, China; 3 State Key Laboratory of Advanced Processing and Recycling of Non-ferrous Metals, Lanzhou University of Technology, Lanzhou, China; 4 College of Computer Science and Technology, Zhejiang University of Technology, Hangzhou, China; 5 Department of Neurosurgery, Lanzhou University Second Hospital, Lanzhou, China

**Keywords:** convolutional neural network, drug-resistant epilepsy, epileptogenic zone localization, stereoelectroencephalography, time-frequency representation

## Abstract

**Introduction:**

Drug-resistant epilepsy (DRE) presents a critical clinical challenge, and accurate epileptogenic zone (EZ) localization is essential for successful surgical intervention. Stereoelectroencephalography (SEEG) is widely applied in clinical practice, but conventional visual inspection and high-frequency oscillations (HFOs) suffer from low efficiency, poor reproducibility, and insufficient representation of complex neural dynamics, failing to improve postsurgical seizure-free rates substantially. To address the limitations, we developed an automated EZ localization framework leveraging ictal SEEG high-resolution time-frequency representation (TFR) and convolutional neural network (CNN).

**Methods:**

Twenty three adult individuals with DRE who achieved Engel Class I surgical outcomes were enrolled in this study. The TFRs of ictal SEEG (0.5–500 Hz) were generated using Short-Time Fourier Transform (STFT), Continuous Wavelet Transform (CWT), and Superlet Transform, then were classified by VGG16/19 and ResNet-18/34/50.

**Results:**

Under subject-independent cross-validation, the optimal HFO-based method achieved an accuracy of 66.10 ± 1.40%, an F1-score of 49.49 ± 2.48% and an AUC of 67.95% ± 2.35%. By comparison, Superlets-VGG19 model achieved significantly better performance: an accuracy of 80.46 ± 4.61%, an F1-score of 79.52 ± 6.88% and an AUC of 80.06 ± 5.23%. Notably, the framework exhibited superior performance in the radiofrequency thermocoagulation (RFTC) sub-cohort, with an accuracy of 82.31 ± 3.41%, an F1-score of 82.56 ± 4.62%, and an AUC of 82.85 ± 3.80%.

**Discussion:**

The proposed framework outperforms conventional HFO-based baselines and provides a reliable and objective tool for clinical EZ localization, and holds substantial optimization potential via integration of complex architectures such as Transformer and Mamba-based models.

## 1 Introduction

Epilepsy is one of the most common neurological diseases globally (Fisher et al., 2017), affecting over 50 million people, with approximately one-third of patients suffering from drug-resistant epilepsy (DRE) (Kwan et al., 2010; Thijs et al., 2019). For these individuals, the most effective curative intervention typically involves the precise localization and surgical removal, disconnection, or targeted ablation of the epileptic focus (Engel et al., 2012; Lüders et al., 2006). SEEG, a minimally invasive modality of intracranial electroencephalogram (iEEG), has emerged as a pivotal diagnostic tool (Talairach and Bancaud, 1973). By recording local field potentials directly from neural tissues, SEEG facilitates the precise delineation of seizure onset zones (EZ) (Kuroda et al., 2025). In clinical practice, the identification of the EZ usually relies on the visual inspection of massive multi-channel SEEG data, a process that is inherently subjective and inefficient (Yang et al., 2024; Asadi-Pooya and Bahrami, 2019). Consequently, there is an urgent demand for automated methodologies capable of recognizing and classifying epilepsy-related SEEG patterns to enhance clinical efficiency (Acharya et al., 2017; Wang et al., 2024d).

Researchers have developed various quantitative methodologies to objectively delineate the EZ. Approaches such as non-linear dynamic analysis (Andrzejak et al., 2001, 2012), functional connectivity modeling and causal brain networks (Jerbi et al., 2009; Wang et al., 2024a,b,c), have been employed to characterize pathological networks. In particular, the analysis of High-Frequency Oscillations (HFOs), encompassing ripples (80–250 Hz) and fast ripples (250–500 Hz), has emerged as a prominent and established method for EZ localization (Staba et al., 2002; Zijlmans et al., 2012). Clinical studies have underscored the critical value of these biomarkers, demonstrating that the resection of HFO-generating areas correlates strongly with seizure freedom, often outperforming traditional spike-based localization (Frauscher et al., 2018). To automate the detection of these critical biomarkers, traditional machine learning (ML) algorithms—such as Support Vector Machines (SVM), Random Forests, and XGBoost—have historically served as the conventional baseline (Wong et al., 2021; Broti et al., 2024). However, these traditional ML-based HFO methods face inherent methodological limitations because they typically rely on 1D handcrafted features extracted directly from the time domain or basic frequency bands, notably lacking the integration of comprehensive Time-Frequency Analysis (TFA) (Zuo et al., 2019). HFOs are exceptionally transient and low-amplitude events that are easily obscured by physiological artifacts or background noise (Shen et al., 2023). Relying solely on manually engineered 1D features lacks the representational capacity to resolve their complex spectro-temporal dynamics. Consequently, these machine learning baselines that do not incorporate time-frequency analysis often struggle to achieve robust detection in noisy clinical environments.

To overcome the representational limitations of 1D handcrafted features, researchers have commonly adopted traditional TFA techniques (Tzallas et al., 2009), such as the Short-Time Fourier Transform (STFT) (Wang et al., 2026), and Continuous Wavelet Transform (CWT) (Shoeibi et al., 2021), as foundational tools to decompose physiological signals into 2D spectral representations, thereby preserving the temporal and spectral dynamics essential for capturing transient HFOs (Ramgopal et al., 2014). For the effective decoding of these high-dimensional spectral images, deep learning approaches, especially Convolutional Neural Networks (CNNs), which excel at interpreting the complex 2D patterns fundamental to HFO detection (Zhang et al., 2021), have gained significant traction to automatically capture latent pathological dynamics. Consequently, researchers have widely adopted signal decomposition techniques to generate spectral images as inputs for CNNs (Schirrmeister et al., 2017; Roy et al., 2019). Nevertheless, a critical bottleneck remains: when attempting to extract transient HFOs, standard TFA methods like STFT reveal their intrinsic limitations. They are bound by the Heisenberg-Gabor uncertainty principle (Gabor, 1946; Wang et al., 2021), which prevents them from simultaneously optimizing time and frequency resolution. This results in the spectral smearing of transient HFOs, often preventing these deep learning models from substantially outperforming traditional ML baselines. Consequently, there is an urgent need for an automated framework capable of systematically integrating high-resolution spectral approaches to fully characterize the complex dynamics embedded in high-sampling-rate intracranial recordings, thereby overcoming current performance bottlenecks and achieving robust diagnostic efficacy.

To address these needs, we present an end-to-end deep learning framework that synergizes multi-resolution time-frequency analysis with deep CNNs for automated EZ localization. Our approach employs a comprehensive suite of spectral decomposition techniques, including STFT, CWT and the Superlet transform (Moca et al., 2021), to process SEEG recordings acquired at elevated sampling frequencies. By visualizing the signals as high-dimensional heatmap-like spectrograms, we aim to capture both broad spectral trends and transient HFO details. These representations serve as inputs for representative CNN architectures VGG16/19 (Simonyan and Zisserman, 2014) and ResNet-18/34/50 (He et al., 2016)), initializing models with pre-trained weights to ensure robust feature extraction and faster convergence.

The primary objective of this research is to realize robust and objective EZ localization through machine learning, establishing a unified benchmark for SEEG-based automated seizure detection. Our strategy aims to exploit the transient high-frequency biomarkers inherent in intracranial data to overcome the poor generalization of traditional HFO algorithms, thereby mitigating human bias and enhancing diagnostic efficiency. The remainder of this paper is organized as follows: Section 2 details the Engel Class I SEEG dataset, the mathematical principles of the high-resolution time-frequency analysis tools, and the specific configurations of the deep learning models. Section 3 evaluates the quantitative performance through subject-independent patient-level cross-validation. Section 4 discusses the advantages of the proposed method and outlines directions for future improvements. Finally Section 5 concludes the study, highlighting its strengths and prospects.

## 2 Materials and Methods

**Figure 1:**
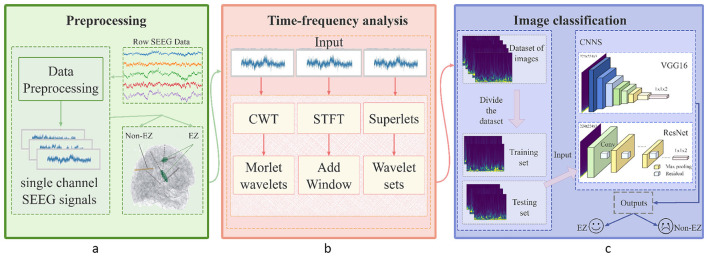
The flowchart of the proposed framework. The architecture consists of three modules: **(a)** Preprocessing: raw SEEG data are decomposed into bipolar signals and spatially labeled as EZ/Non-EZ; **(b)** Time-frequency analysis: 1D signals are converted into 2D spectrograms via STFT, CWT, or Superlet; **(c)** Image classification: CNNs (VGG16/19 and ResNet-18/34/50) are trained on these spectrograms for binary EZ localization.

In this study, we propose an automated SEEG-based framework for EZ localization. We collected high-sampling-rate (2000 Hz) ictal SEEG data from 23 participants with DRE who achieved Engel Class I outcomes following surgery. The spatial ground truth for the EZs in this cohort was strictly defined and verified based on the surgically resected volume. During data preprocessing, the raw SEEG signals were re-referenced to a bipolar montage to effectively mitigate volume conduction and common-mode noise (Zijlmans et al., 2012). Following this data preprocessing step, to extract wideband spectral features, we employed three spectral decomposition techniques, including STFT, CWT, and Superlets, to generate highly detailed temporal and spectral representations. Finally, we employed deep CNNs (VGG and ResNet) to perform binary classification of these representations for the precise identification of EZ channels. The architecture of our proposed framework is shown in Figure 1. Here, we will provide a detailed explanation of the clinical dataset and each methodological module.

### 2.1 Dataset

#### 2.1.1 Basic description

This retrospective study was conducted at the Second Hospital of Lanzhou University, China. The study protocol was approved by the Medical Ethics Committee of the Second Hospital of Lanzhou University (Approval number: 2023A-765). Written informed consent was obtained from all participants or their legal guardians prior to the procedure.

Twenty-three participants with DRE who underwent presurgical SEEG monitoring were enrolled in this study. To ensure the high fidelity of the ground-truth labels, the inclusion criteria were strictly defined as follows: (1) participants who completed a comprehensive multimodal presurgical evaluation (including symptomatology, MRI, and PET) prior to electrode implantation; (2) successful stereotactic implantation of SEEG electrodes designed by the neurosurgical team; (3) availability of continuous SEEG recordings with clear activity of epileptic seizures (yielding 85 seizure episodes), acquired using a Nihon Kohden EEG-1200C system at a high sampling rate of 2,000 Hz; and (4) participants who had undergone targeted surgical resection and achieved complete seizure freedom (Engel Class I) after at least one year of follow-up. The final study sample consisted of 23 participants (seven males and 16 females, age: 18 to 37 years) with optimal surgical prognoses. To directly measure local field potentials and minimize volume conduction, we utilized bipolar derivations (calculating the difference between adjacent contacts on the same electrode shaft) (Jerbi et al., 2009). The EZs were strictly defined by clinicians and neurosurgeons based on the physically resected volume in these seizure-free participants, serving as the ground truth. Other implanted brain zones outside the EZs were defined as non-EZs. The demographic characteristics and clinical information of the participants are detailed in Table 1.

**Table 1:** Detailed clinical demographics and surgical characteristics of the 23 adult participants.

Patient ID	Gender	Age (years)	Age of onset (*y*)	Duration (years)	MRI findings	Number of seizures	Electrodes (L/R)	Number of contacts	Epileptogenic zone localization (EZ)	Surgical procedure
1	M	31	27	4	Positive	5	3/5	94	R. Hippocampus, Amygdala	Resection
2	M	23	3	19	Positive	4	0/9	118	R. Parietal, Temporal Lobes	Combined
3	M	37	36	1	Negative	3	1/5	80	R. Temporal Lobe, Hippo., Amyg.	Resection
4	M	28	20	8	Positive	6	8/0	115	L. Ant. Temporal, Post. Insula	Resection
5	F	28	12	16	Positive	5	8/0	109	L. Temporal Region	Resection
6	M	36	18	18	Positive	2	8/0	122	L. Inf. Frontal Gyrus	Resection
7	M	23	16	7	Positive	3	7/0	96	R. Temporal Lobe	Combined
8	M	30	25	5	Positive	2	9/0	124	L. Ant. Temporal, Med. Temporal	Combined
9	F	26	6	20	Positive	6	0/11	164	R. Medial Temporal, Parietal	RFTC
10	M	20	—	—	Positive	3	8/0	108	L. Parietal Lobe	Resection
11	M	22	7	15	Positive	5	0/9	86	R. Occipital Pole, Cingulate	Resection
12	M	22	20	2	Positive	5	0/9	127	R. Frontal Operculum, Insula	RFTC
13	F	35	16	19	Positive	5	9/0	112	Medial L. Temporal Lobe	RFTC
14	M	28	4	24	Positive	3	9/0	119	L. Ant. Temporal, Med. Temporal	Combined
15	F	31	5	26	Positive	2	8/0	87	L. Precentral Gyrus	RFTC
16	M	27	12	15	Positive	3	1/5	80	R. Temporal Lobe, Hippocampal	Resection
17	F	26	7	19	Negative	3	0/8	108	R. Post. Insula, Parietal Operc.	RFTC
18	M	37	35	2	Positive	3	0/9	118	Medial R. Temporal Lobe	RFTC
19	F	20	18	2	Positive	4	0/9	114	R. Temporo-Parieto-Occipital	RFTC
20	M	18	10	8	Positive	3	0/9	129	R. Parietal, Post. Cingulate	RFTC
21	F	31	21	10	Positive	2	0/11	150	R. Orbitofrontal, Temporal Pole	Combined
22	M	26	1	25	Positive	5	0/6	84	R. Temporal, Frontal Lobes	Resection
23	M	22	9	13	Positive	3	8/0	125	L. Hemisphere	Resection

The “Electrodes (L/R)” column denotes the number of SEEG electrode shafts implanted in the left/right hemisphere, respectively. The “Number of contacts” column indicates the total count of intracranial SEEG recording contacts implanted per patient. Gender: M, male; F, female. MRI (magnetic resonance imaging) findings: Positive = presence of epileptogenic structural abnormalities (lesional); Negative = absence of visible structural abnormalities (non-lesional). R, right hemisphere; L, left hemisphere; Ant, anterior; Post, posterior; Med, medial; Inf, inferior; Hippo, hippocampus; Amyg, amygdala; Operc, operculum; Orbitofr, orbitofrontal; Temp, temporal; Par, parietal; Occ. occipital. Surgical procedures: RFTC, radiofrequency thermocoagulation; Combined, surgical resection combined with RFTC. All participants in this cohort achieved an Engel Class I outcome following the documented procedure.

From each patient's multichannel data, we decomposed the signals into single-channel bipolar recordings, which were then classified into EZ and non-EZ categories. This data processing strategy resulted in a total of 7,858 single-channel samples (2,639 from EZ and 5,219 from non-EZ), transforming EZ localization into a binary classification problem.

#### 2.1.2 Determination process of labels

In this study, the definition of the EZ labels relies on the anatomical correspondence between the SEEG electrode positions and the surgically resected volume, retrospectively validated by clinical outcomes. Our ground truth is derived from the “gold standard” of post-operative seizure freedom. Specifically, we strictly included only participants who achieved Engel Class I outcomes after at least one year of follow-up, ensuring that the resected or ablated volume effectively contained the true epileptogenic zone (Lüders et al., 2006; Rosenow and Lüders, 2001).

Prior to spatial labeling, to mitigate volume conduction and suppress common-mode noise, raw signals were re-referenced using a bipolar montage (Jerbi et al., 2009), calculating the potential difference between adjacent contacts along the same electrode trajectory.

After artifact removal, the label *y*_*j*_∈{0, 1} for each bipolar channel *j* is determined by its spatial relationship with the resection or ablation cavity. Since a bipolar channel is mathematically derived from two adjacent physical contacts, let ${\text{c}}_{j}\in {\mathbb{R}}^{3}$ denote the 3D coordinate of the geometric midpoint between these two contacts. Let $\Omega_{resected}\subset {\mathbb{R}}^{3}$ represent the surgically resected (or RFTC targeted) volume. The labeling function is defined in Equation 1 as:


$$y_{j}=\{\begin{array}{ll} 1(\text{EZ}), & if\;c_{j}\in {\text{Ω}}_{resected}\\ 0(\text{Non}-\text{EZ}), & \text{otherwise} \end{array}$$ (1)

This formulation ensures that positive labels (*y*_*j*_ = 1) correspond to tissue whose removal or ablation resulted in seizure freedom. This objective, coordinate-based protocol was applied uniformly across all 23 participants, avoiding the subjectivity of visual SEEG interpretation and establishing a high-fidelity ground truth for the supervised learning model.

#### 2.1.3 Data quality control and exclusion

To ensure the reliability of the dataset, a standardized quality control protocol was implemented adhering to iEEG preprocessing guidelines (Magnotti et al., 2020). Raw SEEG channels were visually inspected by neurophysiologists to exclude compromised contacts based on the following criteria: (i) mechanical artifacts arising from loose contacts or broken leads; (ii) persistent line noise, specifically 50 Hz interference that remained significant despite notch filtering; and (iii) signal saturation caused by amplifier range overflow.

These compromised channels were removed from the dataset to prevent noise artifacts from confounding the deep learning model. For instance, in Patient P001, six channels were identified as artifactual due to signal instability and were excluded. This selection process ensured that the final dataset consisted of high-quality physiological recordings.

### 2.2 Time-frequency analysis

In this study, we implemented and compared three spectral decomposition paradigms to extract wideband spectral features from the SEEG signals.

#### 2.2.1 Conventional spectral baselines: STFT and CWT

Given that SEEG recordings exhibit non-stationary physiological characteristics, STFT serves as a fundamental baseline for spectral extraction by analyzing quasi-stationary signal segments (Allen and Rabiner, 1977; Beeraka et al., 2022). For the discrete STFT, we implemented the transform using a Hann window (length = 256, overlap = 240) to suppress spectral leakage and generate dense spectral frames. To address the fixed resolution limitation inherent in STFT, the CWT was adopted as a multi-resolution alternative, which dynamically scales and translates a mother wavelet to optimize time-frequency localization (Torrence and Compo, 1998). We employed the Complex Morlet wavelet as the mother wavelet. Its Gaussian-modulated sinusoidal structure exhibits high similarity to the transient oscillatory characteristics of neurophysiological signals, making it effective for extracting SEEG spectral features (Cohen, 2019).

#### 2.2.2 Superlet transform

Standard TFA methods like STFT and CWT are constrained by the Heisenberg-Gabor uncertainty principle, which imposes a traditional trade-off between time and frequency resolution. To optimize this trade-off and improve the detectability of transient pathological features, we employed the Superlet transform (Moca et al., 2021). Superlets achieve time-frequency super-resolution by geometrically combining a set of Morlet wavelets with varying bandwidths, rather than relying on a single, fixed-parameter mother wavelet.

Let Ψ_Λ_ denote a set of Morlet wavelets indexed by their number of cycles *c*. For a specific frequency *f* and a chosen localized order *o*, the set of cycle numbers C is formalized in Equation 2:


$$\begin{array}{c} C=\left\{c_{1},c_{2},\ldots ,c_{o}\right\}\;\text{where}\;c_{i}=c_{min}+\left(i-1\right)\cdot \Delta c \end{array}$$ (2)

Here, *c*_*min*_ is the base cycle number and Δ*c* represents the incremental step. The Superlet transform *SL*(*t, f*) is evaluated as the geometric mean of the multi-cycle CWT responses derived from this combinatorial set, as shown in Equation 3:


$$\begin{array}{c} SL\left(t,f\right)={\left(\overset{o}{\underset{i=1}{\prod }}|CWT_{c_{i}}\left(t,f\right)|\right)}^{1/o} \end{array}$$ (3)

From a signal-processing perspective, this geometric averaging acts as a strict combinatorial filter, sharpening the spectral representation by retaining high magnitudes only if the neurophysiological structure is co-localized across multiple scales. In the context of SEEG processing, this mechanism suppresses broad-spectrum background noise and amplification artifacts, selectively amplifying low-amplitude, localized oscillatory bursts.

To accommodate the wideband pathological continuum of SEEG signals, we utilized the adaptive Superlet scheme, where the transform order *o* varies dynamically as a function of frequency (Moca et al., 2021), defined in Equation 4:


$$\begin{array}{c} o\left(f\right)=o_{min}+\left\lfloor \alpha \cdot \left(f-f_{min}\right)\right\rfloor \end{array}$$ (4)

This adaptive parameterization is highly suited for high-sampling-rate (2000 Hz) intracranial recordings. It maintains high temporal resolution at lower frequency rhythms to preserve discharge onset boundaries, while significantly boosting frequency localization at higher bands, thereby explicitly resolving the spectral microstructure of HFOs without introducing cross-frequency smearing.

### 2.3 Convolutional neural network classification

**Figure 2:**
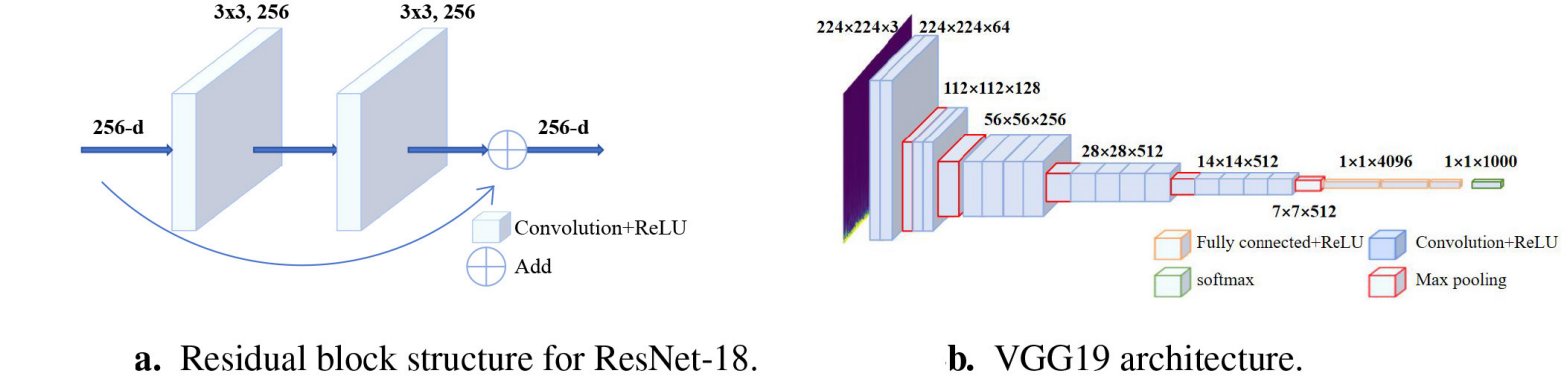
The deep learning architectures employed in this study. **(a)** A fundamental residual block of the ResNet-18 model, featuring identity skip connections (addition) to facilitate gradient flow and mitigate the vanishing gradient problem. **(b)** The layer-by-layer topology of the VGG19 backbone, utilizing stacked 3 × 3 convolutional filters for hierarchical texture feature extraction. ReLU, rectified linear unit.

Deep learning, particularly Convolutional Neural Networks (CNNs), has demonstrated efficacy in extracting hierarchical features from high-dimensional biomedical signals (LeCun et al., 2015). In this study, to evaluate the applicability of the proposed TFR for EZ localization, we selected two representative CNN architectures—VGG and ResNet—as backbone classifiers (Figure 2).

The VGG16/19 architecture employs a homogeneous structure comprising stacks of small 3 × 3 convolutional filters. This design effectively increases network non-linearity while controlling the number of parameters, enabling the learning of local spectral textures indicative of HFOs.

To address the performance degradation often observed in deeper networks, we also evaluated the ResNet. ResNet introduces residual blocks with “skip connections,” which facilitate the direct propagation of gradients. We systematically evaluated three variants: ResNet-18, ResNet-34, and ResNet-50. ResNet-18 serves as a lightweight baseline, while the deeper variants allow us to assess the impact of network depth on classification performance in the context of limited medical data.

Given the limited sample size of clinical datasets compared to large-scale computer vision benchmarks, training deep networks from scratch carries a high risk of overfitting. To address this, we adopted a transfer learning strategy (Tajbakhsh et al., 2016). Both VGG and ResNet models were initialized with weights pre-trained on the ImageNet dataset. This approach allows the models to leverage robust low-level feature extractors (e.g., edge and texture detectors) learned from natural images, which are then fine-tuned to capture the specific spectral morphologies of epileptic discharges.

All generated spectrograms (STFT, CWT, Superlets) were resized to 224 × 224 pixels to match the input dimensions of the pre-trained networks. We did not apply geometric data augmentation techniques (such as random flipping or rotation). Since the axes of spectrograms represent physical dimensions (time and frequency), spatial transformations would violate the signal's spectro-temporal integrity (e.g., flipping would invert time causality or frequency bands), potentially introducing non-physiological artifacts.

We organized the data into EZ and non-EZ classes and employed a strict patient-level Cross-Validation scheme (Kohavi, 1995) to ensure unbiased performance estimation. This method maintains the class distribution in each fold consistent with the entire dataset. The training was implemented using PyTorch on an NVIDIA GPU. We used the Adam optimizer (Kingma and Ba, 2014) with a learning rate of 1 × 10^−4^ and a batch size of 32. Each fold was trained for 130 epochs.

To provide a comprehensive assessment of the model's performance in localizing the epileptogenic zone, we employed six quantitative metrics: Accuracy, Precision, Sensitivity, Specificity, F1-Score, and AUC.

## 3 Results

The proposed deep learning framework was implemented on a local high-performance workstation. The hardware configuration consisted of an Intel Core i9-14900KF processor, NVIDIA GeForce RTX 5090 (32GB). The software environment ran on Windows 10 with Python 3.14, PyTorch 2.11.0.dev20260121+cu130, and CUDA 13.0. The STFT and CWT algorithms were executed using the Scipy and PyWavelets packages, respectively, while the Superlets implementation was adapted from the original author's source code. For classification, all CNN architectures were built using the PyTorch model library. Following the data preprocessing, the final dataset comprised a total of 7,858 single-channel samples. This included 2,639 samples labeled as EZ (positive class) and 5,219 samples labeled as non-EZ (negative class).

To evaluate the model's true clinical generalization and explicitly prevent identity confounding (data leakage) commonly observed in sample-level evaluations, we employed a subject-independent patient-level cross-validation strategy. Under this paradigm, all SEEG segments from a single patient were strictly isolated into either the training or validation set, ensuring the network was evaluated exclusively on entirely unseen participants.

### 3.1 Cross-patient EZ localization

The quantitative performance across the entire patient cohort, evaluated via patient-level 10-Fold cross-validation, is comprehensively summarized in Table 2.

**Table 2:** Cross-patient performance using patient-level.

TFR	Model	Accuracy (%)	Precision (%)	Sensitivity (%)	Specificity (%)	F1-score (%)	AUC (%)
Superlets	ResNet-18	79.02 ± 4.63	79.21 ± 5.87	77.91 ± 8.30	79.77 ± 7.68	78.55 ± 7.03	78.53 ± 5.35
	ResNet-34	77.68 ± 4.70	77.73 ± 5.31	77.52 ± 5.65	78.13 ± 6.02	77.62 ± 5.53	77.45 ± 4.88
	ResNet-50	77.43 ± 4.57	77.34 ± 6.19	77.00 ± 7.38	77.68 ± 7.67	77.17 ± 6.79	77.09 ± 4.60
	VGG16	77.60 ± 4.66	75.80 ± 4.92	77.75 ± 5.63	75.45 ± 5.55	76.76 ± 4.74	75.63 ± 4.52
	VGG19	**80.46** **±** **4.61**	**80.26** **±** **5.92**	**78.79** **±** **8.15**	**80.68** **±** **7.53**	**79.52** **±** **6.88**	**80.06** **±** **5.23**
CWT	ResNet-18	74.71 ± 7.06	74.75 ± 8.68	75.69 ± 7.75	74.37 ± 9.47	75.22 ± 8.06	76.03 ± 7.43
	ResNet-34	75.15 ± 7.19	75.06 ± 9.14	75.22 ± 10.91	73.84 ± 11.25	75.14 ± 10.12	75.65 ± 7.11
	ResNet-50	73.25 ± 7.10	73.47 ± 9.86	73.55 ± 10.47	72.91 ± 11.47	73.51 ± 10.07	74.13 ± 6.92
	VGG16	72.51 ± 6.87	71.84 ± 6.72	73.47 ± 8.15	71.36 ± 6.85	72.65 ± 7.45	72.92 ± 6.99
	VGG19	75.90 ± 6.91	75.66 ± 8.43	76.58 ± 7.60	75.26 ± 9.32	76.12 ± 7.91	76.92 ± 6.76
STFT	ResNet-18	67.62 ± 7.95	70.09 ± 9.64	65.03 ± 12.56	71.21 ± 12.02	67.46 ± 11.05	67.85 ± 7.90
	ResNet-34	68.09 ± 7.93	69.52 ± 9.41	66.55 ± 9.94	70.23 ± 10.58	68.00 ± 9.64	68.76 ± 8.03
	ResNet-50	65.85 ± 8.48	66.72 ± 9.68	64.47 ± 12.77	67.04 ± 11.76	65.58 ± 11.01	65.89 ± 8.33
	VGG16	67.14 ± 8.07	67.97 ± 8.62	65.91 ± 10.08	70.37 ± 10.12	66.92 ± 8.20	67.00 ± 8.38
	VGG19	66.94 ± 7.80	71.08 ± 9.40	65.92 ± 12.41	72.10 ± 11.63	68.40 ± 11.06	67.16 ± 7.75

Bold values indicate the best performance achieved for each respective evaluation metric.

**Figure 3:**
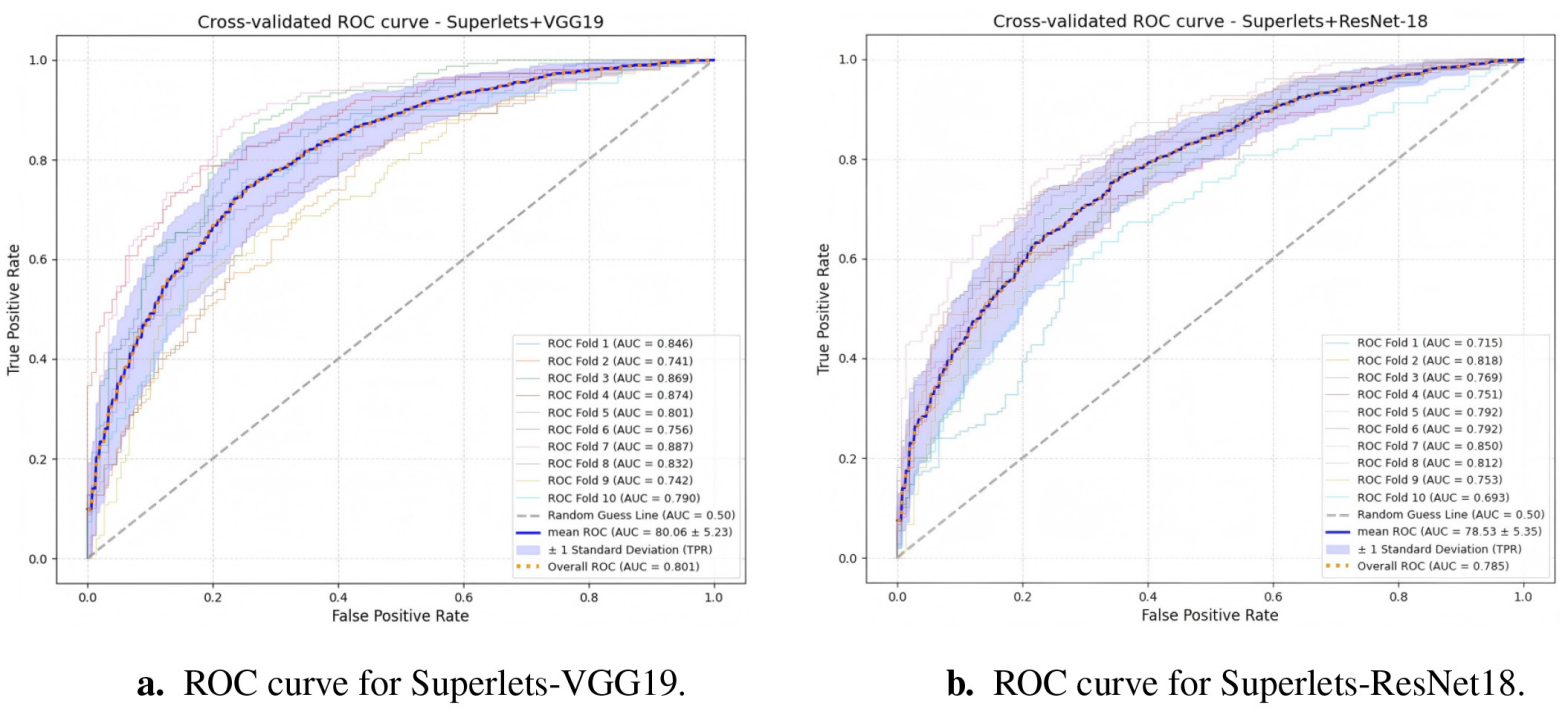
The optimal and sub-optimal ROC curves of the model on the adult dataset. The legend in the lower-right corner has been enlarged for clarity. **(a)** shows the performance of the Superlets-VGG19 model. **(b)** shows the performance of the Superlets-ResNet18 model.

Evidently, the combination of Superlets with the VGG19 architecture achieved the optimal diagnostic performance, yielding an accuracy of 80.46%, precision of 80.26%, sensitivity of 78.79%, specificity of 80.68%, and an AUC of 80.06%. Closely trailing was the ResNet-18 backbone (Accuracy: 78.53%). The optimal and sub-optimal ROC curves of the VGG model are also shown in (Figure 3).

Our comparative analysis reveals a critical insight regarding network depth and feature extraction in medical time-frequency tasks. Excessively deep architectures like ResNet-50 (77.43%) underperformed compared to VGG19 and ResNet-18. This paradox stems from the small dataset sizes in clinical iEEG compared to natural image benchmarks (Raghu et al., 2019). Highly complex networks (e.g., ResNet-50) are prone to overfitting to patient-specific spectral noise in medical domains (Tajbakhsh et al., 2016). Conversely, the dense, uniform 3 × 3 convolutional kernels of VGG19 capture localized, fine-grained texture representations of HFOs effectively without over-parameterization, demonstrating robust inter-patient generalization.

Crucially, the results highlight distinct degradation patterns among the TFR when subjected to inter-patient heterogeneity. Models utilizing STFT suffered severe performance limitations (e.g., STFT + VGG19 achieving only 66.94% accuracy) accompanied by high variance. STFT, constrained by fixed window lengths and the Heisenberg-Gabor uncertainty principle (Gabor, 1946; Bruns, 2004), inherently encodes substantial patient-specific physiological background SEEG. Consequently, STFT models fail to generalize across unseen participants. While CWT offers variable resolution and moderately mitigates this issue (75.90% for VGG19), it still experiences frequency smearing in the critical high-frequency bands where localized pathological ripples reside (Mallat, 1999).

In stark contrast, the Superlets framework maintains remarkable resilience. Experimental results indicate that the Superlets-based approach consistently outperforms conventional CWT and STFT methods. By achieving time-frequency super-resolution, Superlets function as a highly specific filter, isolating patient-independent epileptogenic biomarkers from background noise. This technical advantage prevents the identity confounding observed in baseline methods, ensuring robust and stable generalization across entirely unseen participants. To evaluate granular generalization, individual diagnostic accuracies of the optimal Superlets-VGG19 model for all 23 participants were mapped alongside its overall micro-average accuracy of 80.46% (Supplementary Figure S1). Performance consistently exceeded this overall baseline in patients with highly localized epileptogenic zones, whereas it notably dropped below the average in a few outliers presenting with spatially diffuse, multi-lobar networks.

### 3.2 The high-fidelity RFTC cohort

To further validate our framework on a clinical subset with ultra-precise ground truth, we isolated a sub-cohort of participants who underwent RFTC. Unlike large resective surgeries where the exact boundary of the EZ remains anatomically ambiguous, RFTC lesions provide precise, contact-level confirmation of epileptogenic tissues (Bourdillon et al., 2017). Given the reduced sample size of this specific cohort, we adopted a patient-level 5-Fold CV to ensure an adequate number of participants in each validation fold, providing a smoothing effect against extreme individual outliers while maintaining strict subject independence (Varoquaux, 2018).

As detailed in Table 3, refining the cohort to RFTC participants yielded a performance boost. This overall improvement aligns tightly with the clinical reality that RFTC candidates typically present with highly localized and well-defined epileptogenic networks (e.g., focal cortical dysplasia), providing an ultra-precise, contact-level ground truth (Cossu et al., 2015).

**Table 3:** Cross-patient performance on RFTC cohort using patient-level.

TFR	Model	Accuracy (%)	Precision (%)	Sensitivity (%)	Specificity (%)	F1-score (%)	AUC (%)
Superlets	ResNet-18	79.05 ± 3.43	79.93 ± 4.77	78.43 ± 6.80	80.33 ± 6.38	79.17 ± 6.03	79.29 ± 3.95
	ResNet-34	78.39 ± 3.40	78.40 ± 4.11	77.92 ± 4.75	78.85 ± 4.52	78.16 ± 4.43	79.85 ± 3.28
	ResNet-50	77.60 ± 3.57	77.92 ± 4.79	77.22 ± 6.18	77.98 ± 6.37	77.57 ± 5.29	77.46 ± 3.70
	VGG16	80.45 ± 3.56	78.65 ± 5.62	80.60 ± 4.43	78.30 ± 4.55	79.61 ± 3.34	79.48 ± 3.02
	VGG19	**82.31** **±** **3.41**	**83.51** **±** **4.62**	**81.64** **±** **5.55**	**83.53** **±** **5.43**	**82.56** **±** **4.62**	**82.85** **±** **3.80**
CWT	ResNet-18	76.50 ± 5.76	77.18 ± 7.48	77.49 ± 6.25	76.25 ± 8.37	77.34 ± 8.19	78.35 ± 6.03
	ResNet-34	76.79 ± 5.99	77.23 ± 7.54	77.59 ± 9.61	76.20 ± 9.75	77.41 ± 7.42	77.49 ± 6.21
	ResNet-50	75.04 ± 5.70	75.33 ± 8.56	75.56 ± 9.27	74.57 ± 9.87	75.45 ± 8.97	76.13 ± 5.42
	VGG16	77.13 ± 5.57	75.76 ± 5.52	77.37 ± 7.15	75.30 ± 5.95	76.56 ± 6.05	76.76 ± 5.49
	VGG19	79.63 ± 5.81	79.60 ± 7.13	80.77 ± 6.40	79.36 ± 7.72	80.18 ± 6.91	80.86 ± 5.36
STFT	ResNet-18	66.54 ± 8.65	69.92 ± 8.44	64.35 ± 11.06	70.84 ± 10.92	67.02 ± 9.65	67.97 ± 6.90
	ResNet-34	68.00 ± 7.73	68.82 ± 7.81	66.24 ± 8.64	69.80 ± 9.08	67.51 ± 8.54	68.01 ± 7.13
	ResNet-50	64.69 ± 7.08	66.49 ± 8.38	64.00 ± 11.57	67.03 ± 10.16	65.22 ± 10.01	65.03 ± 8.83
	VGG16	68.86 ± 8.77	68.73 ± 9.42	67.60 ± 8.98	71.36 ± 8.72	68.16 ± 7.20	68.62 ± 8.88
	VGG19	68.94 ± 8.60	72.86 ± 8.10	66.86 ± 10.81	73.73 ± 10.53	69.73 ± 11.66	69.03 ± 8.75

Bold values indicate the best performance achieved for each respective evaluation metric.

Importantly, a comparative evaluation of the network backbones within this high-fidelity RFTC cohort reveals a distinct advantage of the VGG architecture over the ResNet family. The optimal Superlets + VGG19 model achieved the absolute highest performance across all evaluation metrics, peaking at an accuracy of 82.31% and an AUC of 82.85%. This significantly outperformed the residual networks, particularly the deeper ResNet-50 (Accuracy: 77.60%). This performance gap suggests that for the highly localized and pure pathological signals inherent to RFTC ablation cores, the uniform, dense convolutional structure of the VGG architecture is remarkably more effective at capturing fine-grained, high-frequency textural details of HFOs than deep residual learning frameworks.

Analyzing the performance across different TFRs reaffirms the indispensable role of super-resolution analysis in SEEG processing. Even with the highly precise ground truth provided by RFTC lesions, conventional methods like STFT exhibited a pronounced performance ceiling. For instance, the STFT + VGG19 model merely reached an accuracy of 68.94%, trailing its Superlets counterpart by over 13%. This stark contrast emphasizes that the primary bottleneck in automated EZ localization is not solely label noise, but the intrinsic resolution limits of the signal representation. By overcoming the Heisenberg-Gabor uncertainty principle, Superlets act as a “spectral magnifying glass” that clearly delineates transient pathological ripples (Zijlmans et al., 2012) from background physiological rhythms, thereby maximizing the diagnostic utility of the RFTC cohort.

### 3.3 Comparison with traditional ML baselines

To rigorously demonstrate the necessity of integrating Time-Frequency Analysis with deep learning, we compared our optimal framework (Superlets + VGG19) against eleven traditional ML baselines (Table 4), which rely on handcrafted 1D temporal features.

**Table 4:** Performance of traditional HFO-based machine learning baselines.

Model	Accuracy (%)	Precision (%)	Sensitivity (%)	Specificity (%)	F1-score (%)	AUC (%)
AdaBoost	55.29 ± 2.47	39.84 ± 1.17	**61.00** **±** **5.73**	52.35 ± 6.60	48.05 ± 1.14	59.48 ± 1.46
DecisionTree	61.42 ± 1.14	44.15 ± 1.27	50.80 ± 2.27	66.88 ± 2.24	47.21 ± 1.24	58.84 ± 0.94
KNN	61.55 ± 1.37	44.87 ± 1.54	57.43 ± 3.06	63.66 ± 2.14	50.36 ± 1.84	64.41 ± 1.98
LDA	57.49 ± 1.01	40.47 ± 1.07	53.33 ± 3.02	59.63 ± 2.01	46.00 ± 1.60	59.29 ± 1.83
LightGBM	63.80 ± 1.86	47.39 ± 2.27	58.02 ± 3.51	66.78 ± 2.90	**52.13** **±** **2.27**	67.63 ± 1.91
LR	57.44 ± 1.05	40.45 ± 1.14	53.54 ± 3.18	59.44 ± 2.01	46.06 ± 1.72	59.30 ± 1.84
MLP	61.49 ± 2.75	44.96 ± 2.56	55.90 ± 8.32	64.37 ± 7.70	49.47 ± 2.90	64.92 ± 1.88
Naive Bayes	60.48 ± 0.88	40.70 ± 1.66	35.90 ± 3.29	73.12 ± 1.55	38.11 ± 2.47	57.48 ± 1.41
RandomForest	**66.10** **±** **1.40**	**50.10** **±** **2.12**	48.91 ± 3.00	**74.95** **±** **1.16**	49.49 ± 2.48	**67.95** **±** **2.35**
SVM	62.44 ± 1.64	45.21 ± 2.03	49.29 ± 3.27	69.21 ± 2.68	47.12 ± 2.22	63.63 ± 2.21
XGBoost	63.63 ± 1.83	47.10 ± 2.23	56.81 ± 3.04	67.14 ± 1.99	51.49 ± 2.41	67.26 ± 2.07

AdaBoost, adaptive boosting; KNN, K-nearest neighbors; LDA, linear discriminant analysis; LightGBM, light radient boosting machine; LR, logistic regression; MLP, multi-layer perceptron; SVM, support vector machine; XGBoost, extreme gradient boosting. Bold values indicate the best performance achieved for each respective evaluation metric.

The comparative results reveal a performance gap. Among the non-TFA baselines, RandomForest and LightGBM achieved the highest accuracies (66.10 and 63.80%, respectively), trailing our optimal framework (80.46%).

More critically, traditional baselines exhibited high missed detection rates. In EZ localization, sensitivity is an important metric, as missed epileptogenic tissues can lead to surgical failure (Vakharia et al., 2018). The sensitivity of traditional ML models peaked at only 61.00% (achieved by AdaBoost), whereas our proposed Superlets-VGG19 framework achieved a sensitivity of 78.79% (an absolute improvement of over 17%). This empirically confirms that 1D handcrafted features lack the representational capacity to isolate transient, low-amplitude HFOs, leading to suboptimal generalization across unseen participants. In contrast, the 2D time-frequency super-resolution provided by Superlets successfully uncovers these subtle physiological dynamics, thereby establishing a more robust and generalizable diagnostic paradigm (Zuo et al., 2019; Zhao et al., 2022).

**Figure 4:**
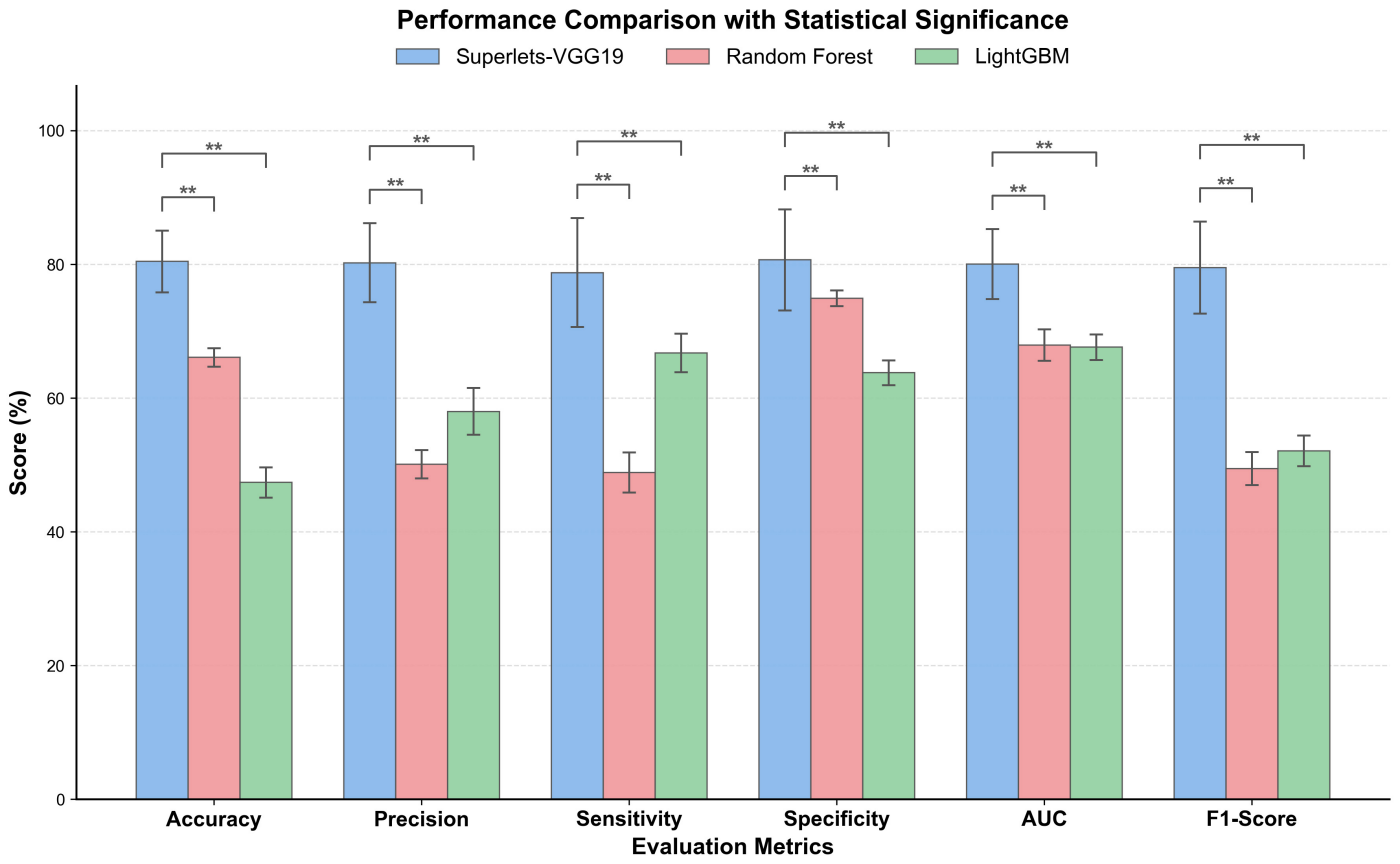
Performance comparison with statistical significance across the 10-fold cross-validation evaluations. The proposed Superlets-VGG19 model is evaluated against the traditional baseline with the highest overall accuracy (Random Forest) and the baseline with the highest F1-score (LightGBM). Statistical significance is calculated using the paired Wilcoxon signed-rank test (***denotes *p* < 0.001; **denotes 0.001 ≤ *p* < 0.01; *denotes 0.01 ≤ *p* < 0.05; ns denotes not significant, *p*≥0.05).

Furthermore, while traditional ML algorithms maintained narrow standard deviations, their overall diagnostic capacity was fundamentally capped at a low performance ceiling due to the restricted expressiveness of 1D temporal features. Conversely, our deep learning framework successfully captured complex 2D spectral dynamics to drastically improve all localization metrics while maintaining robust stability (e.g., a standard deviation of only 4.61% for accuracy), cementing its position as a highly reliable tool for automated EZ localization. To rigorously validate these improvements, paired Wilcoxon signed-rank tests were conducted. As illustrated in Figure 4, the proposed Superlets-VGG19 model demonstrated highly significant enhancements (*p* < 0.01) across key diagnostic metrics compared to both the baseline with the highest overall accuracy (Random Forest) and the baseline with the highest F1-score (LightGBM), proving the performance gains are statistically robust.

### 3.4 Contact-level spatial concordance and clinical mapping

To evaluate the clinical actionability of the proposed framework, we performed a channel-wise spatial mapping between the algorithmic predictions and actual surgical decisions, inspired by recent presurgical validation paradigms (Peng et al., 2023). The detailed spatial overlap for all 23 adult participants is provided in Supplementary Table S1, which juxtaposes the clinically resected or ablated ground truth against the primary epileptogenic hubs identified by our optimal Superlets-VGG19 model.

This micro-level analysis demonstrates that the deep learning model accurately isolates the topological core of the EZ. The macro-level metrics reported in Tables 2, 3 are derived from epoch-level cross-patient evaluation and the channel-level mapping offers a direct and intuitive spatial reference. The high spatial concordance visually corroborates our quantitative findings, confirming that the framework does not indiscriminately approximate expansive surgical boundaries. It provides a highly specific and conservative map of the electrophysiological focus, demonstrating substantial potential to assist neurosurgeons in designing precise ablation trajectories and resective margins.

## 4 Discussion

This study presents an automated framework for EZ localization based on time-frequency analysis and CNNs. While previous studies often treat representations and architectures as interchangeable “black boxes” for algorithmic benchmarking (Roy et al., 2019; Rudin, 2019), this work systematically deconstructs the physical, architectural, and clinical bottlenecks of the EZ localization pipeline. Advancing the field fundamentally beyond standard comparative evaluations, the unique scientific contributions and clinical insights of this study are discussed below.

### 4.1 Exploiting high-frequency oscillations with super-resolution analysis

A primary scientific contribution of this study is establishing the critical necessity of upstream time-frequency super-resolution to mitigate the Heisenberg-Gabor limit. Previous studies on automated EZ localization have made significant progress, yet many were constrained by lower sampling rates or relied on conventional time-frequency methods like STFT. While these approaches effectively capture low-frequency seizure rhythms, they often struggle to resolve HFOs, which are increasingly recognized as specific biomarkers for the epileptogenic zone (Frauscher et al., 2018). Analyzing HFOs poses a fundamental challenge due to the Heisenberg-Gabor uncertainty principle: standard methods cannot simultaneously optimize time and frequency resolution, leading to either spectral smearing or temporal blurring.

Rather than merely substituting one algorithm for another to achieve higher accuracy, this study introduces the Superlet transform to uncover a fundamental representational mechanism. Unlike STFT or CWT, Superlets utilize a set of wavelets with varying bandwidths to achieve “time-frequency super-resolution,” effectively mitigating the intrinsic resolution limitations. Crucially, our cross-patient evaluation revealed a distinct differential degradation among TFA methods. We discovered that STFT suffers severe performance drops in patient-level validation because it is highly vulnerable to overfitting patient-specific background SEEG rhythms. By achieving super-resolution, Superlets act as a highly specific physical filter, isolating genuine HFOs from physiological noise (Moca et al., 2021). This scientific insight demonstrates that optimizing upstream physical signal representation is not merely an algorithmic trick, but a biological prerequisite for extracting universal pathological morphologies and ensuring cross-patient robustness.

### 4.2 Optimal balance between model complexity and generalization

Beyond the conventional objective of blindly searching for state-of-the-art classifiers, this study empirically defines the optimal capacity-generalization boundary for medical CNNs to prevent patient-specific identity confounding. The automated EZ classification algorithm proposed in this study establishes a standardized pipeline that minimizes manual feature engineering.

Although recent trends in computer vision favor ultra-deep architectures (e.g., ResNet-152) or Vision Transformers (Dosovitskiy et al., 2020), our results indicate that VGG16 (a dense classic network) and ResNet-18 (a lightweight residual network) achieved optimal performance, surpassing the more complex ResNet-50. This empirically challenges the prevailing assumption that deeper neural architectures inherently yield superior diagnostic performance in medical time-frequency tasks. This aligns with the consensus that medical datasets, unlike ImageNet, are inherently limited in size and diversity (Tajbakhsh et al., 2016). Overly complex models like ResNet-50 are prone to overfitting, capturing patient-specific acquisition noise rather than generalized physiological features during subject-independent evaluation (Raghu et al., 2019). By purposefully selecting VGG16 or ResNet-18, we struck an optimal capacity-generalization balance, proving that constrained parameter spaces are essential for facilitating reliable clinical deployment.

### 4.3 Surgically validated labeling and the high-fidelity RFTC cohort

A critical aspect of this study is the rigorous definition of the “Ground Truth.” In many previous studies, EZ labels were based on clinicians' pre-surgical hypotheses or included data from participants with failed surgeries, introducing significant label noise (Vafaei and Hosseini, 2025). Evaluating prediction performance using uncertain labels renders the results unreliable.

In contrast, the data used in this study exclusively come from participants who achieved Engel Class I outcomes following surgery. Furthermore, to explicitly address the spatial label noise and anatomical ambiguity inherent in large resective surgeries, we strategically leveraged a high-fidelity validation paradigm utilizing a focal RFTC sub-cohort. RFTC lesions provide a highly precise, contact-level ground truth for epileptogenic tissues, advancing the clinical evaluation methodology beyond the mere approximation of macroscopic surgical boundaries.

On this high-fidelity cohort, our framework achieved an outstanding sensitivity of over 81%. Interestingly, we observed a distinct clinical “scissor difference” where sensitivity significantly outperformed specificity. This aligns perfectly with clinical SEEG reality: while high-frequency pathological signatures at the RFTC ablation core are highly pronounced (ensuring high sensitivity), the propagation of epileptiform discharges into adjacent non-ablated irritative zones can induce false positives, marginally limiting spatial specificity (Jacobs et al., 2010; Roehri et al., 2018). This differential pattern empirically validates that our network successfully learned genuine neurophysiological dynamics rather than artifactual representations.

Furthermore, the superior performance observed in the RFTC sub-cohort compared to the open-resection or combined groups can be fundamentally explained by differences in their spatial extents and anatomical characteristics. Open resections typically involve the removal of large tissue blocks (e.g., standard anterior temporal lobectomy), which encompass both the deep epileptogenic core and superficial neocortical margins (Kohlhase et al., 2021). In contrast, RFTC procedures are inherently highly focal and conservative (Bourdillon et al., 2017). As demonstrated in our clinical cohort, RFTC targets are often well-localized, deep anatomical structures (Cossu et al., 2015). Because RFTC generates discrete, point-like thermal lesions rather than broad regional excisions, the spatial scale of the clinical ground truth strictly matches the localized electrophysiological source identified by our algorithm. This minimal spatial mismatch directly mitigates the label noise introduced by extensive surgical margins, thereby rendering the higher classification accuracy and precision observed in this specific sub-cohort.

### 4.4 Alignment with clinical ground truth: precision vs. sensitivity

This high-fidelity validation directly resolves the prevalent “ground truth paradox” in epilepsy surgery. A fundamental challenge in SEEG-based AI research lies in defining an absolute ground truth. As described in Section 2.1.2, EZ labels were derived from the surgically resected volume in Engel Class I patients. While this approach represents the most rigorous clinical standard, it inherently introduces a “ground truth paradox” (Frauscher et al., 2017; Rosenow and Lüders, 2001). In clinical practice, neurosurgeons routinely resect a safety margin broader than the true epileptogenic core to guarantee seizure freedom. Consequently, the actual seizure onset zone triggering the high-frequency electrophysiological events may be tightly confined to a sub-region, whereas the surgical resection encompasses a more extensive area (Rosenow and Lüders, 2001; Lüders et al., 2006).

This spatial discrepancy profoundly impacts the interpretation of classification metrics. If an algorithm accurately identifies only the highly epileptogenic core, the unselected resected regions are statistically penalized as False Negatives, artificially suppressing the model's theoretical Sensitivity. This phenomenon is quantitatively evident in our cohort evaluation (Table 2), where the optimal Superlets-VGG16 model achieved a robust Precision of 80.26% but a marginally lower Sensitivity of 78.79%. Rather than indicating algorithmic omission, this disparity suggests that the CNN strictly isolates the localized electrophysiological pathology, refusing to overfit to the over-inclusive surgical safety margin.

This observation directly addresses a critical clinical consideration: the relative importance of Precision versus Sensitivity in automated EZ localization. Given the irreversible nature of neurosurgery, False Positives—which could lead to the catastrophic resection of healthy, eloquent cortex—carry a significantly higher clinical risk than False Negatives (Vakharia et al., 2018). Therefore, high Precision is arguably the most valuable metric for presurgical planning. The clinical utility of this precision-driven approach is empirically corroborated by our RFTC sub-cohort results (Table 3). In RFTC procedures, the ablation targets are highly focal and conservative, minimizing the spatial mismatch between the anatomical lesion and the electrophysiological core. Correspondingly, our model's performance peaked in this cohort. Ultimately, instead of merely approximating historical surgical boundaries, the proposed framework provides neurosurgeons with a highly specific, data-driven map of the epileptogenic core, facilitating the transition toward minimally invasive targeted therapies.

### 4.5 Individual patient variance and network heterogeneity

To provide deeper clinical insights into the inter-patient variance observed in Section 3.1, we mapped the individual diagnostic accuracy of all 23 participants (Supplementary Figure S1). The results demonstrate a strong dependency on the spatial characteristics of the epileptogenic networks. Exceptional accuracies (>90%) were exclusively achieved in patients with highly localized pathology, such as strictly confined hippocampal sclerosis (Patient 1) or focal RFTC targets (Patients 9 and 13). In these cases, the epileptogenic core produces pure, easily distinguishable HFO signatures. Conversely, the framework's performance notably declined (< 70%) in challenging outliers presenting with spatially diffuse, multi-lobar involvement (e.g., Patients 2 and 21). This performance degradation is not merely an algorithmic limitation, but rather reflects the inherent electrophysiological challenge of delineating extensive epileptic networks. In such diffuse cases, the spectral boundaries between true primary EZs and secondary propagation zones become highly ambiguous, as high-frequency oscillations can prominently propagate into or co-occur within non-epileptogenic irritative networks (Frauscher et al., 2017). Ultimately, while the proposed framework is highly effective for focal lesions, accurately resolving diffuse epileptic networks remains a critical frontier for computational localization.

### 4.6 Future work and limitations

As a retrospective exploratory study, this work has certain limitations that warrant further improvement. First, regarding the network architecture, the performance drop in ResNet-50 suggests that data scarcity remains a bottleneck. Future work could employ Self-Supervised Learning (SSL) techniques (e.g., MAE) to pre-train models on unlabeled large-scale datasets, potentially improving feature representation in deeper networks (Krishnan et al., 2022). Furthermore, while CNNs excel at extracting localized 2D spectral features, their limited receptive fields constrain the modeling of long-range temporal dependencies (Song et al., 2023). Consequently, the proposed framework offers substantial room for enhancement through the adoption of advanced architectures, such as Transformer and Mamba-based models, to better capture the global temporal dynamics of epileptic discharges (Vafaei and Hosseini, 2025). In addition to high-resolution spectral features, future work could incorporate robust complexity estimators like Cumulative Diversity Pattern Entropy (CDEn) (Wang et al., 2023) as complementary non-linear features, potentially enhancing sensitivity to subtle pathological transitions. Finally, integrating multimodal physiological monitoring, such as combining SEEG with lightweight wearable eye-tracking systems (Yang et al., 2026), could provide comprehensive insights into behavioral semiology during seizures.

Second, the current framework analyzes each channel independently, ignoring the synaptic connectivity between brain regions. However, epilepsy is fundamentally a network disease. Future iterations could incorporate Graph Neural Networks (GNNs) to explicitly model the spatial topology and functional connectivity between SEEG contacts (Li et al., 2021).

Third, this study focused on adult participants. Given that pediatric epilepsy often exhibits different HFO characteristics due to brain development (Cross et al., 2006), future work will extend the dataset to include pediatric cases to investigate the cross-age robustness of our framework. Finally, while our patient-level validation is rigorous, multi-center prospective studies are required to further validate the framework's robustness against varying SEEG acquisition protocols across different institutions.

## 5 Conclusion

In this study, we propose a novel automated deep learning framework for EZ localization using clinical SEEG recordings. First, ictal SEEG signals are acquired and preprocessed. Following this, the acquired signals are decomposed into 2D spectral representations utilizing a suite of time-frequency techniques, namely STFT, CWT, and Superlets, to capture transient HFOs. These representations are then classified using various convolutional neural networks (VGG16/19 and ResNet-18/34/50) to identify the EZ. The experimental results demonstrate that, under patient-level cross-validation, the Superlets-VGG19 and Superlets-ResNet-18 combinations achieve optimal performance, with accuracy rates of 80.46 and 79.02% respectively, outperforming the optimal traditional HFO baseline (Random Forest: 66.10%). Furthermore, targeted evaluation on a RFTC cohort validated the framework's clinical utility, yielding an 82.31% accuracy rate. Ultimately, this objective, data-driven approach offers a powerful tool to assist clinicians in defining precise surgical margins. Future work will focus on incorporating large-scale datasets and complex architectures to enhance model performance and advance clinical translation.

## Data Availability

The raw data supporting the conclusions of this article will be made available by the authors, without undue reservation.
