# Supplementary material for: Localizing the epileptogenic zone in drug-resistant epilepsy based on ictal SEEG high-resolution time-frequency representation
Source: Front Neurosci. 2026 Aug 4;20:1891224. doi: 10.3389/fnins.2026.1891224 (PMC13481549; doi:10.3389/fnins.2026.1891224)
Supplement: Supplementary file 1 [file Data_Sheet_1.pdf]

frontiers

Supplementary Material

1 SUPPLEMENTARY TABLES AND FIGURES

Table S1: **Channel-level spatial concordance between Clinician-defined EZ and ML-classified EZ across the patient cohort.**

Patient ID	Clinician-defined EZ Channel IDs (Surgical Ground Truth)	ML-classified EZ Channel IDs (Superlets-VGG16 Model)	Overlapping Channel IDs (True Positives)
P001	11-14, 19, 21-22, 25-27	11-12, 15, 19, 21-22, 25-26	11-12, 19, 21-22, 25-26
P002	1-19, 21-42, 50-57, 80-87, 104-111	1-18, 20, 22-41, 48-49, 51-56, 81-86, 104-110	1-18, 22-41, 51-56, 81-86, 104-110
P003	1-8, 11-14, 19, 21-27, 34-37, 39-40, 45-50	1-6, 9, 11-13, 19, 21-25, 34-36, 39-40, 46-49	1-6, 11-13, 19, 21-25, 34-36, 39-40, 46-49
P004	12-18, 36-37, 39-74	10, 13-17, 36-37, 40-73, 76	13-17, 36-37, 40-73
P005	1-6, 11-19, 21-22, 25-37, 61-73, 86-89, 96-101	2-5, 8, 12-18, 21-22, 26-36, 59, 62-72, 86-88, 97-100	2-5, 12-18, 21-22, 26-36, 62-72, 86-88, 97-100
P006	11, 18-19, 21, 23-24, 35-37, 39-44, 62-72, 112-118	11, 15, 18-19, 21, 23-24, 34-36, 40-43, 63-71, 113-117	11, 18-19, 21, 23-24, 35-36, 40-43, 63-71, 113-117
P007	7-14, 33-37, 39-54, 57-62, 80-82	8-13, 33-36, 40-53, 55, 58-61, 80-81	8-13, 33-36, 40-53, 58-61, 80-81
P008	1-19, 21-31, 39-42, 51-56, 64-74, 91-94, 107-112	2-18, 22-30, 38-41, 48, 52-55, 65-73, 91-93, 108-111	2-18, 22-30, 39-41, 52-55, 65-73, 91-93, 108-111
P009 (RFTC)	11-13, 29-33, 48-55, 68-69, 73-79, 89-92, 104-106, 116-117, 120-121, 132-133, 149-152	11-13, 29-32, 48-54, 68-69, 73-78, 89-91, 95, 104-106, 116-117, 120-121, 132-133, 149-151	11-13, 29-32, 48-54, 68-69, 73-78, 89-91, 104-106, 116-117, 120-121, 132-133, 149-151
P010	18-19, 21, 28-35, 47-56, 68-74, 78-90	15, 18-19, 21, 29-34, 48-55, 69-73, 79-89	18-19, 21, 29-34, 48-55, 69-73, 79-89
P011	11, 18-19, 21, 23-24, 35-37, 39-44, 62-72, 112-118	11, 18-19, 21, 23-24, 35-36, 40-43, 50, 63-71, 113-117	11, 18-19, 21, 23-24, 35-36, 40-43, 63-71, 113-117
P012 (RFTC)	1-5, 18-19, 26-28, 38-42, 52-55, 78-88	1-4, 18-19, 26-28, 31, 38-41, 52-54, 78-87	1-4, 18-19, 26-28, 38-41, 52-54, 78-87
P013 (RFTC)	5-9, 11-14, 23-24, 28-30	4-7, 11-13, 16, 23-24, 28-30	5-7, 11-13, 23-24, 28-30
P014	1-32, 70-77, 101-117	2-31, 71-76, 85, 102-116	2-31, 71-76, 102-116
P015 (RFTC)	5-8, 11-14, 25-28, 33-37, 43-46, 56-59, 62-64, 72-76	5-7, 11-13, 25-27, 33-36, 43-45, 56-58, 62-64, 67, 72-75	5-7, 11-13, 25-27, 33-36, 43-45, 56-58, 62-64, 72-75
P016	1-19, 21-37, 39-42, 51-60	2-18, 22-36, 39-41, 46, 52-59	2-18, 22-36, 39-41, 52-59
P017 (RFTC)	28-29, 40-52	27-29, 33, 40-51	28-29, 40-51
P018 (RFTC)	1-4, 11-12, 30-33, 47-58, 63-68, 79-81, 85	1-3, 11-12, 30-32, 40, 47-57, 63-67, 79-81, 85	1-3, 11-12, 30-32, 47-57, 63-67, 79-81, 85
P019 (RFTC)	4, 7-9, 21-22, 25-26, 36-37, 43-50, 61-66, 95-99	4, 7-9, 15, 21-22, 25-26, 36-37, 43-49, 61-65, 95-98	4, 7-9, 21-22, 25-26, 36-37, 43-49, 61-65, 95-98
P020 (RFTC)	4-9, 14-19, 21-22, 28-42, 68-91, 96-101, 112-119	4-9, 13-18, 21-22, 28-43, 55, 68-90, 96-100, 112-118	4-9, 14-18, 21-22, 28-42, 68-90, 96-100, 112-118
P021	1-8, 21-22, 25-33, 55-78, 99-114	2-7, 21-22, 26-32, 40, 56-77, 100-113	2-7, 21-22, 26-32, 56-77, 100-113
P022	8-10, 13-16, 22-27, 43-52, 57-70	8-9, 13-15, 20, 23-26, 44-51, 58-69	8-9, 13-15, 23-26, 44-51, 58-69
P023	62-77, 86-109	60-75, 80, 87-108	62-75, 87-108

Note: The Clinician-defined EZ Channel IDs represent the surgical volume serving as the ground truth, extracted from clinical annotations. The ML-classified EZ Channel IDs represent the predictions generated by our optimal framework (Superlets-VGG16) under strict cross-patient validation. Patients marked with (RFTC) underwent Radiofrequency Thermocoagulation. It is important to note that this table specifically summarizes the primary contiguous electrophysiological hubs mapped at the anatomical channel level to provide an intuitive spatial reference. Because the machine learning framework evaluates independent temporal signal segments during cross-patient testing, this micro-level channel

listing primarily reflects the localized topological core of maximum seizure-onset density, rather than an exhaustive channel-wise aggregation of all discrete epoch-level classification outputs.

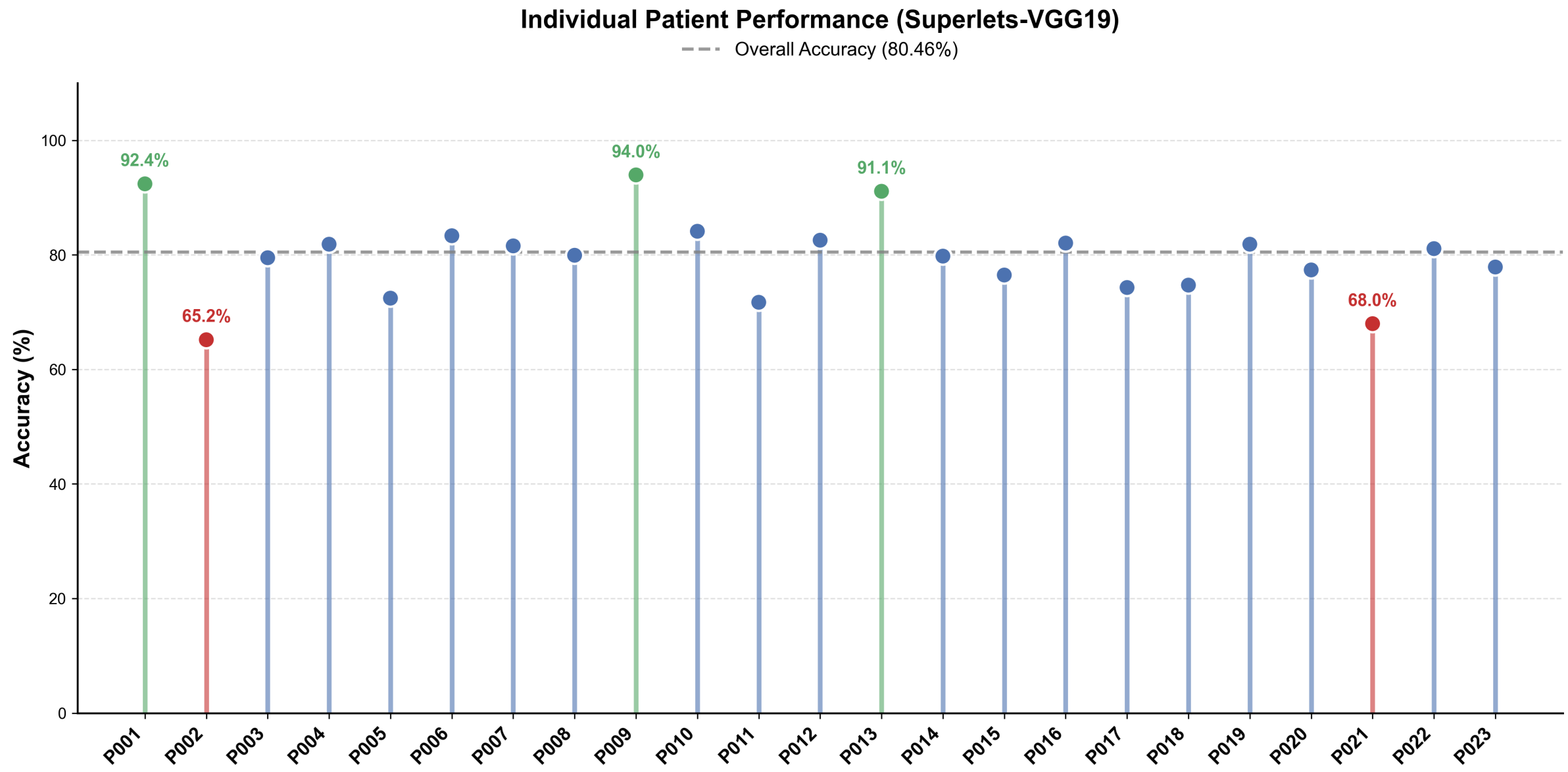

Figure S1: Individual patient performance across the 23 adult participants using the optimal Superlets-VGG19 framework. The overall micro-average accuracy (80.46%) is denoted by the dashed gray line.
